# Visual classification of three computed tomography lung patterns to predict prognosis of COVID-19: a retrospective study

**DOI:** 10.1186/s12890-021-01813-y

**Published:** 2022-01-03

**Authors:** Daisuke Yamada, Sachiko Ohde, Ryosuke Imai, Kengo Ikejima, Masaki Matsusako, Yasuyuki Kurihara

**Affiliations:** 1 Department of Radiology, St. Luke’s International Hospital, 9-1 Akashi-cho, Chuo-ku, Tokyo, 104-8560 Japan; 2 Graduate School of Public Health, St. Luke’s International University, 9-1 Akashi-cho, Chuo-ku, Tokyo, 104-8560 Japan; 3 Department of Pulmonary Medicine, Thoracic Center, St. Luke’s International Hospital, 9-1 Akashi-cho, Chuo-ku, Tokyo, 104-8560 Japan

**Keywords:** COVID-19, Computed tomography, Respiratory function, Retrospective study

## Abstract

**Background:**

Quantitative evaluation of radiographic images has been developed and suggested for the diagnosis of coronavirus disease 2019 (COVID-19). However, there are limited opportunities to use these image-based diagnostic indices in clinical practice. Our aim in this study was to evaluate the utility of a novel visually-based classification of pulmonary findings from computed tomography (CT) images of COVID-19 patients with the following three patterns defined: peripheral, multifocal, and diffuse findings of pneumonia. We also evaluated the prognostic value of this classification to predict the severity of COVID-19.

**Methods:**

This was a single-center retrospective cohort study of patients hospitalized with COVID-19 between January 1st and September 30^th^, 2020, who presented with suspicious findings on CT lung images at admission (n = 69). We compared the association between the three predefined patterns (peripheral, multifocal, and diffuse), admission to the intensive care unit, tracheal intubation, and death. We tested quantitative CT analysis as an outcome predictor for COVID-19. Quantitative CT analysis was performed using a semi-automated method (Thoracic Volume Computer-Assisted Reading software, GE Health care, United States). Lungs were divided by Hounsfield unit intervals. Compromised lung (%CL) volume was the sum of poorly and non-aerated volumes (− 500, 100 HU). We collected patient clinical data, including demographic and clinical variables at the time of admission.

**Results:**

Patients with a diffuse pattern were intubated more frequently and for a longer duration than patients with a peripheral or multifocal pattern. The following clinical variables were significantly different between the diffuse pattern and peripheral and multifocal groups: body temperature (*p* = 0.04), lymphocyte count (*p* = 0.01), neutrophil count (*p* = 0.02), c-reactive protein (*p* < 0.01), lactate dehydrogenase (*p* < 0.01), Krebs von den Lungen-6 antigen (*p* < 0.01), D-dimer (*p* < 0.01), and steroid (*p* = 0.01) and favipiravir (*p* = 0.03) administration.

**Conclusions:**

Our simple visual assessment of CT images can predict the severity of illness, a resulting decrease in respiratory function, and the need for supplemental respiratory ventilation among patients with COVID-19.

## Background

Since the first outbreak of coronavirus disease 2019 (COVID-19), numerous patients have been admitted to the hospital with respiratory symptoms. Clinical manifestations of COVID-19 range from asymptomatic or mild upper respiratory tract disease to severe interstitial pneumonia with respiratory failure, requiring oxygen support and intubation [1–5]. More than a year after the worldwide COVID-19 outbreak, there are no signs of the pandemic abating.

Numerous clinical and imaging markers of disease severity have been reported. Among imaging markers, computed tomography (CT) provides the most sensitive radiological technique for the diagnosis of COVID-19, revealing diffuse lung alterations, ranging from ground-glass opacity to consolidation [5–10]. In addition, different radiological lung patterns are manifested over the course of the disease. Research using quantitative methods to evaluate lung CT images to derive various image analysis scores has suggested the possibility of predicting the severity of the disease [10–14]. However, there are limited opportunities in clinical practice to use these quantitative image analysis approaches [11, 12, 14]. To address this limitation, we recently conducted a single-center retrospective study of patients with COVID-19 at St Luke’s International Hospital, Tokyo, to investigate if a simple visual assessment of CT images could predict the severity of COVID-19. We identified three patterns of pneumonia findings based on visual analysis of CT images, which could be associated with disease severity. Our primary objective in this study was to investigate the reliability of the classification of these patterns and their utility in predicting the clinical outcomes of COVID-19.

## Methods

### Study oversight

**Fig. 1:**
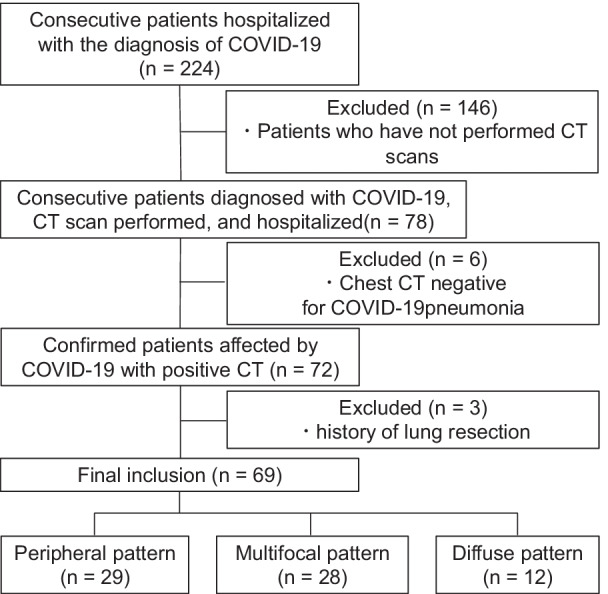
Flowchart depicting the patient selection process. COVID-19: coronavirus disease 2019, CT: computed tomography

This retrospective study was approved by the institutional review board of St. Luke’s International Hospital (20-R220). Informed consent was waived owing to the retrospective study design. We used our hospital’s electronic medical record database to retrospectively identify 224 consecutive patients who had been admitted to the hospital with a diagnosis registered as “COVID-19” or “suspected of COVID-19” from January 2020 to September 2020. Only patients with a COVID-19 diagnosis confirmed by reverse transcription-polymerase chain reaction were selected. Patients who did not have a CT scan at admission, as well as those with a history of lung resection, were excluded. Patients with no abnormal lung findings on CT were also excluded. Ultimately, 69 consecutive patients with COVID-19 were included in our analysis (Fig. 1).

### CT protocols

In each patient, the whole lung CT was performed under static conditions during an end-inspiratory hold whenever possible. CT imaging was performed using either a 256-detector scanner (Revolution CT, GE Healthcare) or 64-detector scanners (Optima CT660, GE Healthcare). The parameters for CT examinations performed on the Revolution CT unit were as follows: 120-kV tube voltage, 50 to 650 mA tube current, 80 mm collimation, 0.992 pitch, 320 mm field of view (FOV), and 512 × 512 matrix. Parameters for the Optima CT660 unit were as follows: 120-kV tube voltage, 50–560 mA tube current, 40 mm collimation, 0.984 pitch, 320 mm FOV, and 512 × 512 matrix. An unenhanced scan was obtained for all patients. The dose length product was 525.54 ± 290.7 mGy-cm, with a volume CT dose index of 11.21 ± 3.75 mGy.

### CT findings

**Fig. 2:**
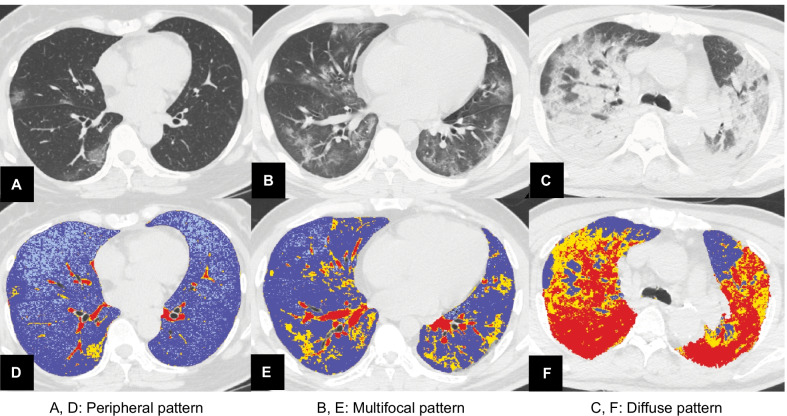
Computed tomography (CT) images for the three patterns of COVID-19 pneumonia. The pulmonary opacities are classified into peripheral, multifocal, and diffuse patterns. **a** In the peripheral pattern, parenchymal opacification appears in the inner peripheral zone. **b** In the multifocal pattern, parenchymal opacification is apparent in the central and peripheral regions. **c** The diffuse pattern reveals generalized pulmonary involvement, with regional inhomogeneity. **d**–**f** Semi-automated segmentation using Thoracic VCAR software (GE Healthcare, USA). Blue areas represent normal lung parenchyma in the -501, -900 HU interval; light blue areas represent hyperinflated lung in the -901, -1000 HU; yellow areas represent poorly aerated lung in the -500, -100 HU interval; and red areas represent non-aerated lung in the 100, -100 HU interval. COVID-19, coronavirus disease 2019; HU, Hounsfield unit; VCAR, Volume Computer-Assisted Reading

Pulmonary opacities were classified into peripheral, multifocal, and diffuse patterns according to the classification by Akira et al. [15]. Parenchymal opacification predominantly appeared in the subpleural peripheral zone in the peripheral pattern. In contrast, multiple parenchymal opacifications were apparent in both central and peripheral regions in the multifocal pattern. Diffuse patterns revealed generalized pulmonary involvement, with or without heterogeneity (Fig. 2). Further CT findings included consolidation, linear opacities, reversed halo sign, and crazy-paving sign [5, 6, 8–10, 13]. The definitions of these CT findings were based on the uniform terms for thoracic imaging by the Fleishner society [16]. We also assessed the number of affected lobes.

All CT assessments were reviewed by two radiologists (DY and KI), with 5 and 8 years of experience, respectively, who were blinded to clinical patient data. These two radiologists were involved with the original CT examinations in the clinical setting. For the study, CT assessment was performed without any clinical information, at least 3 months after the initial clinical assessment. The CT images were randomized for assessment to prevent recall bias. Discrepancies in classification of CT findings between the two radiologists were resolved by consensus.

### Quantitative analysis

The dataset was anonymized and exported to a dedicated segmentation suite for medical image computing (GE Healthcare, USA), equipped with a semi-automated segmentation algorithm (Thoracic Volume Computer-Assisted Reading software). The software performed a first-pass automated segmentation. Lung volumes were then manually perfected using three-dimensional tools, such as spherical brushes or erasers. A complete segmentation included both lungs with interstitial structures, segmentary vessels, and bronchi. The major pulmonary arteries and bronchi, all mediastinal structures, eventual pleural effusion, and lung masses (e.g., tumors, fungal disease) were excluded. We extracted the lung volumes and calculated the percentage of the total volume affected, according to different Hounsfield unit (HU) intervals, into non-aerated (NNL) (% NNL, density between 100 and − 100 HU), poorly aerated (PAL) (% PAL, − 101 to − 500 HU), normally aerated (NAL) (% NAL, − 501 to − 900 HU), and hyperinflated (− 901 to − 1000 HU) [17]. The additional “compromised lung” (% CL) volume was calculated as the sum of % PAL and % NNL (− 500 to 100 HU) (Fig. 2). The authors in charge of the segmentation (T.S. and D.U.) were unaware of the laboratory and clinical parameters or hospitalization outcomes of patients. Any discrepancies were resolved by a consensus between the two radiologists. The principal investigator reviewed and confirmed all segmentations before data entry. We recorded the time required to complete each analysis.

### Data sources

We used chart review to obtain clinical information, including physical examination findings and laboratory data at the time of admission; and the clinical course after hospitalization. Moreover, we collected data for the date of onset, the date of CT imaging, the time between onset and CT scan, the date of hospitalization, the duration of hospitalization, the presence of tracheal intubation, the duration of tracheal intubation, history of intensive care unit (ICU) admission, and death. The following clinical information was collected immediately after hospitalization: respiratory rate, oxygen saturation (SpO2), partial pressure of oxygen (PaO2), pulse rate, systolic blood pressure, diastolic blood pressure, body temperature, white blood cell count, lymphocyte count, neutrophil count, platelet count, C-reactive protein (CRP), lactate dehydrogenase (LDH), Krebs von den Lungen-6 antigen (KL-6), D-dimer, and if the patient was on steroids, heparin, or favipiravir. The steroids administered were dexamethasone 6.6 mg/day or methylprednisolone 1 mg/kg/day intravenously. Heparin was administered by continuous infusion of heparin Na (10,000 units/day) or subcutaneous injection of heparin Ca (5,000 units) twice daily. Favipiravir was administered at a dose of 1800 mg twice daily on day 1, and 800 mg twice daily on day 2 and thereafter.

### Statistical analyses

To evaluate the reproducibility of the classification, we calculated the interobserver reliability for each finding using the Cohen kappa value. The following ratings were used to interpret the kappa value: poor, < 0.40; moderate, 0.40–0.59; good, 0.60–0.80; and excellent, > 0.80.

The association between the three CT patterns and compromised lung (% CL) was evaluated using the Kruskal–Wallis test. The association between the three CT patterns and ICU admission, intubation management, and death was assessed using the chi-squared test. The Kruskal–Wallis test was used to compare the duration of hospitalization, duration of intubation, and time from the onset to CT scan between the three CT patterns. Univariate and multivariate logistic regression analyses were performed to evaluate the association between each CT finding and intubation. The correlation between the CT patterns and clinical information was calculated. Parametric Fisher analysis of variance (ANOVA) was used for between-pattern comparison of clinical variables with a normal distribution (pulse rate, systolic blood pressure, diastolic blood pressure, body temperature, neutrophil count, platelet count, CRP, and D-dimer); Kruskal–Wallis test was used for variables with a non-normal distribution (respiratory rate, SpO2, PaO2, white blood cell count, lymphocyte count, neutrophil count, LDH, KL-6, Acute Physiology and Chronic Health Evaluation-II score, and sequential organ failure assessment [SOFA] score). Differences in the use of steroids, heparin, and favipiravir between the three patterns were evaluated using the chi-squared test. Subsequently, we conducted the Bonferroni test and Mann–Whitney U test as a post-hoc test. Univariate and multivariate analyses were performed on the CT findings. All statistical analyses were performed using Stata 16.1 (StataCorp LP, TX, United States). A *p *value < 0.05 was considered statistically significant.

## Results

Pretreatment CT images for each patient were classified into one of the following three patterns: diffuse, multifocal, and peripheral. There was good interobserver reproducibility in the classification of images (κ = 0.74).

**Fig. 3:**
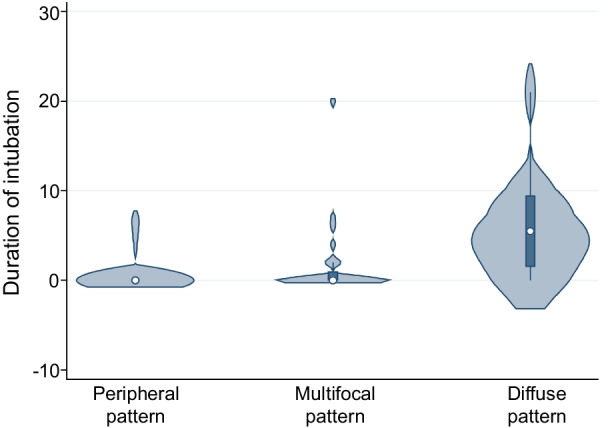
Violin plots for the three CT patterns and duration of intubation. The line represents 95% CI, the box represents the interquartile range, and the point is representative of the median. Density plot width indicates the frequency. There was a significant difference between the three CT patterns and duration of intubation (Kruskal–Wallis test, *p* < 0.0001). On the Mann–Whitney U test, there were significant differences between the peripheral and diffuse patterns and between the multifocal and diffuse patterns (*p* = 0.003 and *p* = 0.001, respectively). CT, computed tomography

There were differences in the rate of ICU admission, tracheal intubation, and death between the three patterns (*p* = 0.07, *p* < 0.01, and *p* = 0.06, respectively), with the difference in the rate of tracheal intubation alone being significant. Further, each pattern was associated with a different duration of hospitalization, duration of tracheal intubation, and the time from the onset to CT scan (*p* = 0.09, *p* < 0.01, and *p* = 0.80, respectively); the difference in the duration of tracheal intubation alone was significant (Fig. 3). These variables also showed significant differences between the peripheral and diffuse patterns and between the multifocal and diffuse patterns (*p* < 0.01 and *p* < 0.01, respectively).

**Fig. 4:**
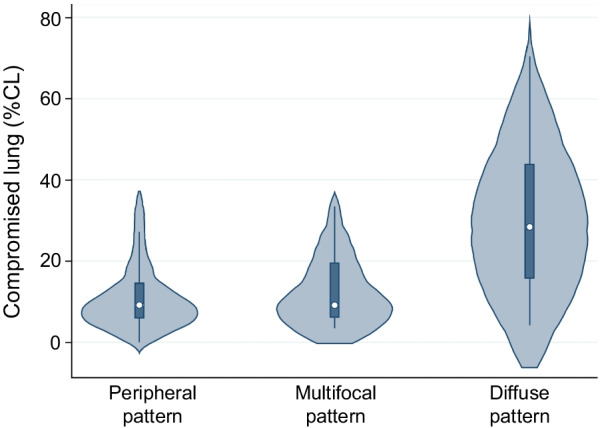
Violin plots for the three CT patterns and compromised lung (% CL). There was a significant difference between the three CT patterns and compromised lung (% CL) (Kruskal–Wallis test, *p* = 0.003). On the Mann–Whitney U test, there were significant differences between the peripheral and diffuse patterns and between the multifocal and diffuse patterns (*p* = 0.001 and *p* = 0.002, respectively). CT, computed tomography

There was a significant difference between the three CT patterns and volume of the compromised lung (% CL) (*p* < 0.01; Fig. 4). On post-hoc analysis, there was a significant difference in % CL between the peripheral and diffuse patterns and between the multifocal and diffuse patterns (*p* < 0.01, respectively).

There were significant differences in temperature, CRP, and D-dimer, lymphocyte count, neutrophil count, LDH, KL-6, and administration of steroid and favipiravir between the three CT patterns (*p* < 0.05 each, Table 1).

**Table 1:** Demographic and clinical data stratified according to the three CT patterns (peripheral, multifocal, and diffuse)

	Total (n = 69)	Peripheral pattern (n = 29)	Multifocal pattern (n = 28)	Diffuse pattern (n = 12)	*p* value
*Characteristics*					
Male sex (%)	21 (30%)	11 (38%)	7 (25%)	3 (25%)	0.52
Age (years)	57.8 (47–72)	55.2 (42–69)	58.9 (49–72.5)	61.8 (44–75)	0.43
*Hemodynamic parameters*					
Pulse rate (per min)	87.17 (80–96)	87.11 (80–98)	86.12 (77–94)	89.83 (83.5–100.5)	0.75
Systolic BP (mmHg)	122.82 (113–130)	120.11 (112–130)	127.72 (119–134)	118.01 (107–129)	0.06
Diastolic BP (mmHg)	76.71 (68–86)	73.61 (66–82)	80.52 (70–90)	75.13 (62–86.5)	0.16
Body temperature (°C)	37.5 (36.9–38)	37.5 (36.8–37.8)	37.7 (37.2–38.25)	37.1 (36.45–37.6)	0.04
*Respiratory characteristics*					
Respiratory rate (breaths per min)	20.71 (16–24)	18.91 (16–20)	22.12 (16–27.5)	21.83 (18–24.5)	0.10
SpO2 (%)	95.59 (95–97)	95.9 (95–98)	96.1 (95–97.5)	93.8 (91.5–96.5)	0.08
PaO2/FiO2 ratio	230.71(134.4-270.8)	270.72(198.6-294.1)	227.92(141.8-290.1)	202.01(103-255)	0.19
PaCO2 (mmHg)	35.51(33.7-37.1)	34.82(33.7-35.4)	35.53(33.7-37.1)	36.11(33.7-37.1)	0.81
*Biomarkers*					
WBC (×1000) (/μL)	5.97 (4–7.1)	5.81 (3.4–7.5)	5.62 (3.8–6.55)	7.31 (5.6–8.7)	0.10
Lymphocyte count (/μL)	889.95 (598–1190)	1210.11 (852.6–1602)	1003.43 (560.5–978.8)	672.42 (328–1190)	0.01
Neutrophil count (/μL)	3859.45 (2252–4902.8)	3478.31 (1788.4–4650.5)	3597.91 (2532.7–4438.2)	5842.24 (3062.7–7526.4)	0.02
CRP (mg/dL)	7.82 (1.43–11.92)	5.83 (0.47–9.87)	7.59 (1.905–11.71)	13.32 (9.41–17.76)	0.01
LDH (IU/L)	320.15 (213–388)	247.60 (184–302)	314.83 (233.5–396.5)	511.91 (358–629)	<0.01
KL6 (U/mL)	408.28 (214–526.5)	244.61 (190–234)	293.41 (206–352)	813.90 (473.5–1094.5)	<0.01
Platelet (×10^3^/μL)	192.59 (158–229)	191.70 (149–242)	187.82 (160–217.5)	207 (146–229)	0.66
D-dimer (μg/mL)	4.18 (0.6–1.8)	1.50 (0–1.6)	0.99 (0–1.1)	16.8 (1.5–25.9)	<0.01
*Therapeutic agents*					
Steroid	48 (69.57%)	15 (52%)	12 (43%)	12 (100%)	0.01
Heparin	48 (69.57%)	17 (59%)	22 (79%)	9 (75%)	0.24
Favipiravir	14 (20.29%)	2 (7%)	7 (25%)	5 (42%)	0.03
*Severity scores*					
APACHE-II score	16.78 (13–19)	15 (11.5–18)	16.3 (13–19)	19.3 (15.5–23)	0.37
SOFA score	5.48 (3–7)	4.5 (3–5.5)	5.1 (3–6)	7 (5–9)	0.17

APACHE-II, Acute Physiology and Chronic Health Evaluation-II; BP, blood pressure; CRP, C-reactive protein; CT, computed tomography; LDH, lactate dehydrogenase; KL6, Krebs von den Lungen-6 antigen; PaO2, partial pressure of oxygen; SOFA, sequential organ failure assessment; SpO2, oxygen saturation; WBC, white blood cell

We performed separate statistical analyses for patients with and without tracheal intubation (Table 2), with a comparison of their clinical parameters. Respiratory rate, white blood cell count, lymphocyte count, neutrophil count, CRP, LDH, KL-6, SpO2, D-dimer, steroid, favipiravir, and SOFA were significantly correlated with intubation (*p* < 0.05; Table 3).

**Table 2:** Comparison of CT findings between patients with and without intubation (n = 69)

	Intubation (−) (n = 50)	Intubation (+) (n = 19)	*p *value
Peripheral pattern (%)	26 (52%)	3 (16%)	< 0.01
Multifocal pattern (%)	21 (42%)	6 (32%)	0.43
Diffuse pattern (%)	3 (6%)	9 (47%)	< 0.01
Compromised lung (% CL)	12.36 (5.98–15.67)	23.86 (11.75–32.49)	< 0.01
Affected lobes	4.76 (4–6)	5.74 (6–6)	0.01
GGO (%)	49 (98%)	19 (100%)	0.54
Consolidation (%)	36 (72%)	18 (95%)	0.04
Linear opacities (%)	46 (93%)	18 (95%)	0.70
Reversed halo (%)	1 (3%)	0 (0%)	0.54
Crazy-paving (%)	24 (48%)	14 (74%)	0.06
Emphysema (%)	4 (8%)	3 (16%)	0.34
Fibrosis (%)	1 (2%)	0 (0%)	0.54
Fatty liver (%)	20 (40%)	11 (58%)	0.18

CT, computed tomography; GGO, ground-glass opacity

**Table 3:** Demographic and clinical data and intubation status

	Intubation (−) (n = 50)	Intubation (+) (n = 19)	*p* value
*Characteristics*			
Male sex (%)	17 (34%)	4 (21%)	0.30
Age (years)	57.4 (35–82)	58.9 (47–72)	0.75
*Hemodynamic parameters*			
Pulse rate (per min)	85.8 (79–96)	90.8 (80–101)	0.19
Systolic BP (mmHg)	122.42 (112–130)	124.01 (116–132)	0.67
Diastolic BP (mmHg)	76.6 (67–86)	77.1 (68–87)	0.88
Body temperature (°C)	37.42 (36.9–37.8)	37.7 (37.3–38)	0.14
*Respiratory characteristics*			
Respiratory rate (breaths per min)	19.7 (16–20)	23.5 (18–28)	< 0.01
SpO2 (%)	96.30 (95–98)	93.74 (93–97)	< 0.01
PaO2/FiO2 ratio	265.8(167.4–334.7)	199.3(119.7–234.4)	0.09
PaCO2 (mmHg)	35.5(32.1–38.0)	35.5(33.7–37.1)	0.59
*Biomarkers*			
WBC (× 1000) (/μL)	5.6 (4–6.5)	6.9 (3.6–8.9)	0.01
Lymphocyte count (/μL)	1174.4 (780–1350)	683.7 (413–936)	< 0.01
Neutrophil count (/μL)	3375.5 (2029–4526)	5103.81 (2657–6675)	0.02
CRP (mg/dL)	5.47 (0.97–9.87)	13.63 (9.08–10.24)	< 0.01
LDH (IU\L)	272.95 (199–307)	436.89 (342–510)	< 0.01
KL6 (U/mL)	274.47 (209–289)	559.93 (221–885)	0.04
Platelet (× 10^3^/μL)	193.94 (158–238)	189.26 (146–222)	0.77
D-dimer (μg/mL)	1.52 (0–1.15)	10.10 (0.9–6.3)	< 0.01
*Therapeutic agents*			
Steroid (%)	31 (62%)	17 (89%)	0.03
Heparin (%)	33 (66%)	15 (79%)	0.30
Favipiravir (%)	7 (14%)	7 (37%)	0.04
*Severity scores*			
APACHE-II score	16.21 (13–18)	17.12 (13–22)	0.73
SOFA score	4.11 (2–5)	6.29 (4–8)	0.05

APACHE-II, Acute Physiology and Chronic Health Evaluation-II; BP, blood pressure; CRP, C-reactive protein; KL6, Krebs von den Lungen-6 antigen; LDH, lactate dehydrogenase; PaO2, partial pressure of oxygen; SOFA, sequential organ failure assessment; SpO2, oxygen saturation; WBC, white blood cell

**Fig. 5:**
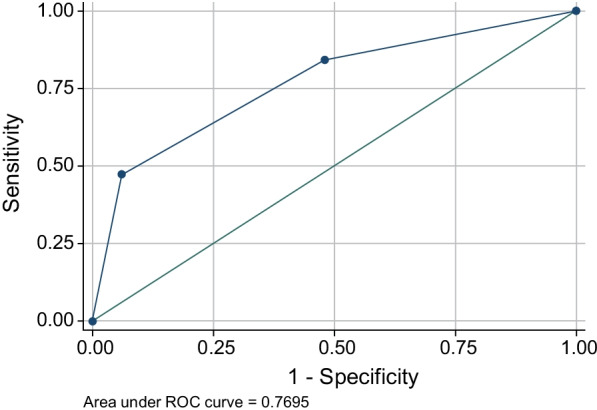
Results of the ROC curve analysis for the three patterns in predicting intubation. The three CT patterns predicted the tracheal intubation, with an area under the ROC curve of 0.77. CT, computed tomography; ROC, receiver operating characteristic

On univariate logistic regression analysis, three CT pattern and % CL were significant (CT pattern, *p* < 0.01; odds ratio [OR], 3.52; [95% confidence interval [CI], 1.17–4.12]; % CL, *p* < 0.01; OR, 2.85; [95% CI 0.02–0.12]). On multivariate analysis, three CT patterns alone were retained as an independent predictive factor of intubation (*p* = 0.02; OR, 2.34; [95% CI 0.2–2.27]; Table 4). The three CT patterns predicted the tracheal intubation, with an area under the receiver operating characteristic (ROC) curve of 0.77 (Fig. 5).

**Table 4:** Univariate and multivariate analyses of CT findings

	Univariate			Multivariate		
	Odds ratio	95% CI	*p* value	Odds ratio	95% CI	*p* value
Three CT pattern	3.52	1.17–4.12	< 0.01	2.34	0.2–2.27	0.02
Compromised lung (% CL)	2.85	0.02–0.12	< 0.01	1.09	− 0.25 to 0.09	0.28
Affected lobes	1.87	0.02–1.06	0.06	− 0.08	− 0.62 to 0.57	0.94
Consolidation	1.81	0.16–4.05	0.07	1.06	− 1.05 to 3.54	0.29
Crazy paving	1.87	0.05–2.27	0.06	0.68	− 0.92 to 1.89	0.50

*CT* computed tomography, *CI* confidence interval

## Discussion

The findings of this study show that patients with a diffuse pattern on pretreatment lung CT had a higher and prolonged requirement for intubation. Furthermore, we tested quantitative CT analysis as an outcome predictor for COVID-19 using a semi-automated method. In univariate logistic regression analysis, three CT patterns and quantitative CT analysis were significant. However, only the three CT patterns were retained as an independent predictor of tracheal intubation in the multivariate logistic regression analysis, with patients with a diffuse CT pattern being at the highest risk and requiring prolonged duration of intubation. COVID-19 pneumonia has an extremely variable prognosis [17–21]. While 80% of patients are either asymptomatic or have mild symptoms, 20% develop severe or profound disease and eventually die [22–25]. CT imaging of the lungs plays an important role in the care of patients infected with severe acute respiratory syndrome coronavirus-2 (SARS-CoV-2), with the prognostic value of CT evaluated in several studies [9–14, 18–21]. However, most of these studies are from China, Europe, and the United States, with no information available for patient cohorts in Japan [22–26]. In addition, several researchers have used image analysis software to quantify CT findings, making these impractical in daily clinical practice [25–27]. Practicality in clinical practice was our motivation to develop and evaluate the prognostic value of our visually-based assessment of CT lung findings (peripheral, multifocal, and diffuse) in a hospital patient cohort in Tokyo, Japan, a city with a particularly high SARS-CoV-2 prevalence. COVID-19 pneumonia shares a similar pathogenesis to acute exacerbation of interstitial pneumonia. Volume loss because of alveolar collapse is the primary cause of traction bronchiectasis in COVID-19 pneumonia. However, the extent of involved alveoli and mucosa may expand with disease progression. In addition, inflammation-related damage to the bronchial walls may lead to fibrosis, bronchiectasis, and bronchial wall thickening. Thus, COVID-19 is associated with acute respiratory distress syndrome and may produce CT findings similar to acute exacerbation of interstitial pneumonia [17]. Akira et al. reported that the prognosis of acute exacerbation in interstitial pneumonia could be predicted by classifying findings on lung CT images into peripheral, multifocal, and diffuse patterns [15]. In our study, we applied the same classification to patients with COVID-19 and evaluated if these three patterns were predictive of patients’ clinical course. By simply classifying the CT images of a COVID-19 patient into three patterns, we can predict the requirement and duration of intubation for that patient, allowing us to quickly and easily allocate optimal medical resources to the patient. In addition, this study revealed that the requirement for intubation can be predicted through our method as efficiently as, or better than, through quantitative analysis of CT images, indicating the possibility that the prognosis of COVID-19 patients can be predicted to some extent by visual judgment of CT images even in institutions that cannot introduce software for CT analysis.

We also compared the clinical variables in three different CT patterns. As a result, the following clinical variables were significantly different between the diffuse pattern and peripheral and multifocal groups: body temperature, lymphocyte count, neutrophil count, c-reactive protein, lactate dehydrogenase, Krebs von den Lungen-6 antigen, D-dimer, and steroid and favipiravir administration. These results suggest that the more extensive the abnormal findings in the lungs, the more severe the systemic over-inflammatory response in COVID-19. The laboratory features presented by this study could be attributed to respiratory failure, septic shock, and/or multiple organ dysfunction or failure compatible with the acute respiratory distress syndrome course of severe and critical COVID-19 types [17, 25–29]. Our simple visual assessment of CT images revealed the possibility of predicting systemic cytokine storm in COVID-19 patients. The identification of prognostic factors at an early stage of the disease could help guide clinicians in providing an optimal treatment path based on patient-specific characteristics, as well as predict more precisely where medical resources are most required.

The reason for the lack of an association between mortality and the length of hospital stay and the three CT patterns is unclear. Nonetheless, the study population presumably included some of the earliest patients following the pandemic, and the treatment methods were inconsistent, thereby resulting in a lack of correlation between mortality and the length of hospital stay. Another reason is that the aggressive use of steroids and favipiravir in patients with severe COVID-19 may have prevented a significant difference in mortality. We intend to collect more data in the future to clarify the association between CT findings and mortality. The limitations of our study should be acknowledged in the interpretation of our results. First, this was a single-center retrospective study with a small sample size. Second, only Japanese patients from the city of Tokyo were included. As such, the risk of bias related to viral factors, such as host factors and genomic variation, cannot be discounted. We do note the benefit of a single center for ensuring a uniform assessment of images. Third, cases in the early stages of the COVID-19 pandemic in Japan were included in this study, and treatment methods were not consistent. Fourth, although we instructed all patients to breathe in as much as possible when CT was performed, there was a possibility that patients with poor respiratory status did not have sufficient inhalation volume. This may have affected the quantitative analysis of CT. Fifth, our hospital is one of the facilities that preferentially accepts critically ill patients with COVID-19 in our area. Therefore, we accept the patients with severe disease at our hospital and all the patients who underwent CT scan were subject to oxygen inhalation on admission. This may cause the selection bias on this study. Sixth, we did not follow the time course of the changes in CT findings in each case. Lastly, the inclusion criteria were limited to patients with pulmonary lesions on the initial CT scan; patients with no abnormalities on the initial CT scan were not included.

## Conclusions

Our simple visual assessment of CT images can predict the severity of illness, a decrease in respiratory function, and the need for supplemental respiratory ventilation among patients with COVID-19.

## Data Availability

The datasets during and/or analyzed during the current study available from the corresponding author on reasonable request.

## Abbreviations

CL: Compromised lung
CRP: C-reactive protein
FOV: Field of view
ICU: Intensive care unit
KL-6: Krebs von den Lungen-6 antigen
LDH: Lactate dehydrogenase
PaO2: Partial pressure of oxygen
SpO2: Oxygen saturation
%NNL: Non-aerated
%PAL: Poorly aerated
%NAL: Normally aerated
